# Overview of Ongoing Clinical Trials on Radioembolization

**DOI:** 10.1007/s00270-022-03270-4

**Published:** 2022-10-02

**Authors:** Matthias P. Fabritius, Jens Ricke

**Affiliations:** Department of Radiology, University Hospital, LMU Munich, Marchioninistr. 15, 81377 Munich, Germany

The role and data driven basis for radioembolization remains ill-defined with recent phase III randomized trials negative for patients with hepatocellular carcinoma (HCC), and only a single second line trial in metastatic colorectal cancer (CRC) meeting its primary endpoint progression-free survival (EPOCH, NCT01483027) [1]. Few studies are currently underway to define and/or clarify the role of radioembolization. The search term “radioembolization OR SIRT OR TheraSphere OR TARE” (status: Aug. 2022), revealed 44 clinical trials at clinicaltrials.gov, of which only 25 are currently recruiting. However, additional active trials can be found in registries such as the European Union´s register EudraCT (2 additional active trials) [2] or national such as the German DRKS (3 additional active trials) [3]. Stratifying ongoing clinical trials as published in PubMed or clinicaltrials.gov, the following categories can be defined: (a) clinical trials evaluating the efficacy of SIRT in different tumor biologies metastatic to or primaries of the liver; as the most important subgroup, evaluation of combination therapies with systemic treatment, predominantly combining with checkpoint inhibition, including basic research seeking immune-modulating mechanisms and proof in tissue or blood; (b) assessing new technologies such as Holmium-166 for SIRT; and (c) new approaches (e.g., highly selected patient group) and indications for SIRT, also outside the liver.

The only phase III trial ongoing likely is STOP-HCC (NCT01556490), with advanced HCC patients receiving sorafenib ± radioembolization. Primary endpoint is overall survival, results are expected for end of 2022. However, this concept may come late given most recent developments in systemic HCC treatment shifting to immune checkpoint inhibition [4, 5]. Considering the presumptive immunomodulatory effect of radioembolization such combination therapies might be the future domain of radioembolization, which is supported by the results of a recently published phase II study from Singapore (NCT03033446, radioembolization + Nivolumab) [6] as well as by the promising preliminary results of a phase I (NCT03099564, radioembolization + Pembrolizumab) [7] and phase II study (NCT03380130, radioembolization + Nivolumab) [8]. Therefore, phase II trials such as IMMUWIN (NCT04522544; randomized, radioembolization or transarterial chemoembolization [TACE] combined with Durvalumab/Tremelimumab, primary endpoint objective response rate [ORR], completion 2024), SOLID (NCT04124991; single arm, radioembolization + Durvalumab, time-to-progression, completion 2022) or ROWAN (NCT05063565; randomized, radioembolization alone vs. radioembolization + Durvalumab/Tremelimumab, ORR, completion 2026) are of utmost interest for positioning radioembolization in HCC future treatment strategies. Recently evolved, the ZUGSPITZE trial, may add mechanistic as well as conceptual data through an extensive basic research program (EudraCT 2020-003,925-42, Sponsor LMU München, randomized three arm study, radioembolization standard vs. personalized dose + Durvalumab/Tremelimumab vs.checkpoint first followed by radioembolization on demand, endpoint ORR, completion estimated 2025). Further Phase II single arm studies combining radioembolization and immune checkpoint inhibition target cholangiocellular carcinoma (IMMUWHY, NCT04238637), CRC (SIRTCI, NCT04659382; iRE-C, NCT04108481) or neuroendocrine tumors (NCT03457948). Finally, the only active clinical cancer trial for non-liver indications seems to be POEM (DRKS00021723), a phase I/II trial assessing safety and efficacy as a composite endpoint when using the bronchial arteries to target primary or secondary lung malignancies. Completion is expected 2023. Table 1 provides a selected overview of currently ongoing trials with comments regarding the crucial characteristics of each study.

**Table 1:** Selected overview of ongoing trials

Status	Register number	Short title	Official study title	Tumor	Primary endpoint	Study start date	Comment
*active, recruiting*	NCT02936388	SirTac	A Randomized Phase II Trial of RE With Yttrium-90 (SIRT) in Comparison with Transarterial Chemoembolisation With Cisplatin (TACE) in Patients With Liver Metastases From Uveal Melanoma	Uveal melanoma LM	PFS	01/2016	Only trial on Uveal melanoma metastases
	DRKS00009744	AROMA	Distant effects of radioembolization of hepatic malignancies on nonirradiated tumor tissue	CLM		03/2016	Basic research/ tumor biology
	NCT03059030		Yttrium-90 RE for Cirrhosis-Associated Thrombocytopenia			03/2017	New indication
	NCT05227482	DOSEY90	Accurate Dosimetry and Biomarkers Improve Survival in HCC Patients Treated With Resin 90 Yttrium-Microspheres: a Randomized Trial	HCC	OS	04/2018	Trial on dosimetry
	NCT03457948		A Pilot Study of Pembrolizumab and Liver-Directed Therapy or Peptide Receptor Radionuclide Therapy for Patients With Well-Differentiated NETs and Symptomatic and/or Progressive Metastases	NET LM	BOR	08/2018	RE + immune checkpoint inhibition
	NCT04362436	ArTisaN	A Phase II Assessment of the Safety and Efficacy of TheraSphere® SIRT in the Treatment of Metastatic (Liver) NETs	NET LM	ORR, AE incidence	01/2019	Trial on NET metastases
	NCT04238637	IMMUWHY	Phase II Study of Immunotherapy With Durvalumab (MEDI4736) or Durvalumab and Tremelimumab, Both Combined With Y-90 SIRT in Advanced Stage ICC	ICC	ORR	11/2019	RE + immune checkpoint inhibition
	DRKS00021723	POEM	Study on feasibility and dose finding of SIRT with Y-90 microspheres in patients with malignant primary or secondary lung tumors	Any primary or secondary lung malignancy		06/2020	New indication
	NCT04108481	iRE-C	Immunotherapy Combined With Yttrium-90 RE in the Treatment of Colorectal Cancer With Liver Metastases	CLM	MTD	10/2020	RE + immune checkpoint inhibition
	NCT04659382	SIRTCI	A Prospective, Multicenter, Open-label, Phase II Study to Evaluate Efficacy and Safety of SIRT Plus Xelox, Bevacizumab and Atezolizumab in Patients With Liver-dominant Metastatic Colorectal Cancer	CLM	PFS	10/2020	RE + immune checkpoint inhibition
	NCT04541173		A Randomized Phase II Study of Atezolizumab and Bevacizumab With Y-90 TARE in Patients With Unresectable HCC	HCC	PFS	11/2020	RE + immune checkpoint inhibition
	NCT04522544	IMMUWIN	A Phase II Study of Immunotherapy With Durvalumab (MEDI4736) and Tremelimumab in Combination With Either Y-90 SIRT or TACE for Intermediate Stage HCC With Pick-the-winner Design	HCC	ORR	12/2020	RE + immune checkpoint inhibition
	NCT04605731		A Phase Ib Study of Durvalumab (Medi4736) and Tremelimumab Following RE in Patients With Unresectable Locally Advanced HCC	HCC	Overall response rate, DLT, AE Incidence	08/2021	RE + immune checkpoint inhibition
	NCT04339036	CapTemY90	UPCC 04,219 Phase 2 Study of Capecitabine-Temozolomide(CapTem) With Yttrium-90 RE in the Treatment of Patients With Unresectable Metastatic Grade 2 NETs	NET LM	hepatic PFS	10/2021	Trial on NET metastases
	NCT05265208	SIROCHO	A Multicenter Open-label Randomized Controlled Prospective Phase II Study Evaluating the Efficacy of SIRT (Yttrium-90 Glass Microspheres) Combined With Capecitabine in the Neoadjuvant Setting of Operable ICC	ICC	Frequency of subjects with adequate surgical margins	02/2022	Trial on ICC
	DRKS00009916	SWARM	Systemic release of growth factors after RE of hepatic malignancies	Any primary or secondary hepatic malignancy		03/2022	Basic research/ tumor biology
	NCT05063565	ROWAN	An Open-Label, Prospective, Multi-Center, Randomized Clinical Trial to Evaluate The Efficacy and Safety Of TheraSphere Followed by Durvalumab (Imfinzi®) With Tremelimumab vs. TheraSphere Alone For HCC	HCC	ORR, DoR	08/2022	RE + immune checkpoint inhibition
*active, not recruiting*	NCT01556490	STOP-HCC	A Phase III Clinical Trial of Intra-arterial TheraSphere in the Treatment of Patients With Unresectable HCC	HCC	OS	03/2012	Only phase III trial
	NCT02807181	SIRCCA	Prospective, Multicenter, Randomized, Controlled Study Evaluating SIR-Spheres Y-90 Resin Microspheres Preceding Cisplatin-gemcitabine (CIS-GEM) Chemotherapy vs. CIS-GEM Chemotherapy Alone as First-line Treatment of Patients With Unresectable ICC	ICC	Survival at 18 months	01/2017	First-line unresectable ICC
	NCT03099564	HCRN GI15-225	A Pilot Study of Pembrolizumab in Combination With Y90 RE in Subjects With Poor Prognosis HCC With Preserved Liver Function	HCC	PFS	03/2017	RE + immune checkpoint inhibition
	NCT03812562		Pilot Study of Nivolumab in Combination With Therasphere (Yttrium-90) for Treatment of HCC With Intent for Resection	HCC	Recurrence rate	02/2019	RE + immune checkpoint inhibition
	NCT04124991	SOLID	A Single-arm, Open-label, Safety and Efficacy Study of RE With Yttrium-90 Microspheres in Combination With Durvalumab (MEDI4736) in Locally Advanced and Unresectable HCC	HCC	TTP	06/2020	RE + immune checkpoint inhibition
*not yet recruiting*	2020–003,925-42	Zugspitze	A randomized open-label phase II study on the effect of durvalumab and tremelimumab combined with personalized SIRT, standard-dose SIRT or immunotherapy followed by on-demand loco-regional SIRT in non-resectable HCC patients	HCC	ORR	10/2021	RE + immune checkpoint inhibition and dosimetry
	NCT05114148	iHEPAR	Individualized Dosimetry for Holmium-166-RE in Patients With Unresectable HCC; a Multi-center, Interventional, Non-randomized, Non-comparative, Open Label, Early Phase II Study	HCC	Rate of unacceptable toxicity	11/2021	Holium-166, dosimetry
	NCT05092880	CAIRO7	RE in Elderly/Fragile Patients With Unresectable Livermetastases of Colorectal Cancer	CLM	PFS	11/2021	Highly selected patient cohort
	NCT05327738		A Phase II Study to Evaluate the Efficacy and Safety of Y-90, Atezolizumab and Cabozantinib Among Patients With Unresectable and Locally Advanced HCC	HCC	Proportion of progression-free participants	06/2022	RE + immune checkpoint inhibition
	NCT05303467	FRONTIER	A Feasibility Study to Evaluate the Safety of the TheRaSphere GliOblastoma (GBM) Device iN PaTIEnts With Recurrent GBM	GBM		07/2022	New indication
	NCT05377034	STRATUM	A Multinational, Double-blind, Placebo-Controlled, Parallel Randomized Arms, Phase II Trial to Compare Safety and Efficacy of SIRT (Y-90 Resin Microspheres) Followed by Atezolizumab Plus Bevacizumab vs. SIRT Followed by Placebo in Patients With Locally Advanced HCC	HCC	BOR	07/2022	RE + immune checkpoint inhibition
	NCT05315687		Safety and Efficacy of RE of Metastatic Breast Cancer to the Liver as a 2nd/3rd Line Therapy	BCLM	PFS	07/2022	Trial on breast cancer metastases
	NCT05422690		A Phase II Trial of Induction Gemcitabine, Cisplatin and Nab-Paclitaxel Triplet Chemotherapy Followed by Gemcitabine, Cisplatin and RE for the Treatment of Locally Advanced Unresectable ICC	ICC	ORR	07/2022	Trial on ICC
	NCT05451862	HOMIE-166	Holmium-166 Transarterial RE in Unresectable, Early Stage HCC; a Prospective, Single-arm, Open Label, Multicenter Phase II Study	HCC	ORR	12/2022	Holium-166
	NCT05195710		Preoperative Y-90 RE for Tumor Control and Future Liver Remnant Hypertrophy in Patients With Colorectal Liver Metastases	CLM	Feasibility and safety	05/2023	New indication

AE, adverse event; BCLM, Breast cancer liver metastases; BOR, Best observed overall response rate; CLM, Colorectal liver metastases; DLT, Dose-limiting toxicity; DoR, Duration of Response; GBM, Glioblastoma; HCC, Hepatocellular carcinoma; ICC, intrahepatic cholangiocarcinoma; LM, liver metastases; MTD, maximum tolerated dose; NET, neuroendocrine tumor; ORR, Objective response rate; PFS, Progression-free survival; RE, radioembolization; TTP, Time to progression

In summary, only a small number of clinical trials are currently active, almost all limited to small Phase II concepts. Given the lack of comprehensive data on the clinical benefit of radioembolization, emerging data clearly will not suffice to secure radioembolization as integral part of future treatment strategies and guidelines.
